# Characterization of the visceral and neuronal phenotype of 4L/PS-NA mice modeling Gaucher disease

**DOI:** 10.1371/journal.pone.0227077

**Published:** 2020-01-13

**Authors:** Victoria Schiffer, Estibaliz Santiago-Mujika, Stefanie Flunkert, Staffan Schmidt, Martina Farcher, Tina Loeffler, Irene Schilcher, Maria Posch, Joerg Neddens, Ying Sun, Jan Kehr, Birgit Hutter-Paier

**Affiliations:** 1 QPS Austria GmbH, Neuropharmacology, Grambach, Austria; 2 Pronexus Analytical AB, Bromma, Sweden; 3 Division of Human Genetics, Cincinnati Children’s Hospital Medical Center, Cincinnati, Ohio, United States of America; 4 Department of Pediatrics, University of Cincinnati College of Medicine, Cincinnati, Ohio, United States of America; Weizmann Institute of Science, ISRAEL

## Abstract

Gaucher disease is caused by a deficiency in glucocerebrosidase that can result in non-neuronal as well as neuronal symptoms. Common visceral symptoms are an increased organ size, specifically of the spleen, and glucosylceramide as well as glucosylsphingosine substrate accumulations as a direct result of the glucocerebrosidase deficiency. Neuronal symptoms include motor deficits and strong alterations in the cerebellum. To evaluate the effect of new compounds for the treatment of this devastating disease, animal models are needed that closely mimic the human phenotype. The 4L/PS-NA mouse as model of Gaucher disease is shown to present reduced glucocerebrosidase activity similar to human cases but an in-depth characterization of the model was still not performed. We therefore analyzed 4L/PS-NA mice for visceral alterations, motor deficits and also neuronal changes like glucocerebrosidase activity, substrate levels and neuroinflammation. A special focus was set at pathological changes of the cerebellum. Our results show that 4L/PS-NA mice have strongly enlarged visceral organs that are infiltrated by enlarged leukocytes and macrophages. Furthermore, animals present strong motor deficits that are accompanied by increased glucosylceramide and glucosylsphingosine levels in the brain, astrocytosis and activated microglia in the cortex and hippocampus as well as reduced calbindin levels in the cerebellum. The latter was directly related to a strong Purkinje cell loss. Our results thus provide a detailed characterization of the 4L/PS-NA mouse model over age showing the translational value of the model and validating its usefulness for preclinical efficiency studies to evaluate new compounds against Gaucher disease.

## Introduction

Gaucher disease (GD) is a sphingolipidosis and thus belongs to the large group of lysosomal storage diseases. GD is autosomal recessively inherited and caused by a deficiency in glucocerebrosidase (GCase) resulting in an accumulation of its substrate glucosylceramide (GlcCer) as well as glucosylsphingosine (GlcSph). In most patients, the disease is caused by a point mutation in the GCase gene *GBA1* and thus far more than 400 different *GBA* mutations are known according to the Human Genome Mutation Database (HGMD). Although the disease is today assumed to have a phenotypic spectrum from very weak to severe, historically 3 disease types can be distinguished. While the by far most common form of GD, Type 1, is characterized by almost pure visceral symptoms, Type 2 and 3 are characterized by visceral and neuronal symptoms. Patients carrying GBA1 mutations have an increased risk of developing Parkinson disease and Dementia with Lewy bodies [1, 2]. Currently, there are two different types of treatments available to patients: enzyme replacement therapy (ERT) and substrate reduction therapy (SRT). ERT is based on the provision of GCase that diseased cells are lacking. SRT is based on the reduction of excessive cytoplasmic GlcCer (for review see [3]). Both treatment types result in a slow but sufficient effect on visceral symptoms, but no evidence exists for an effect on neuronal symptoms [4]. Future drug developments are therefore ought to focus on compounds that are effective against neuronal GD symptoms. To test these new compounds *in vivo*, rodent animal models that properly mimic neuronal GD pathology are utilized. There are several GD mouse models available that are based on different disease aspects like depleted or reduced prosaposin expression [5, 6], *Gba1* deletion [7, 8] or *Gba1* point mutations [9, 10]. Major issues of these models are severely reduced life spans or weak pathological features, making these models impracticable for treatment studies. The 4L/PS-NA mouse model by Grabowski and colleagues combines two disease aspects by expressing the *Gba1*^V394L/V394L^ mutation and additionally reduced prosaposin/saposins levels [11]. The model presents visceral and neuronal symptoms and a life span of approximately 22 weeks. The GCase and saposin activity of 4L/PS-NA mice are strongly reduced, resulting in early clusters of lipid storage cells in several visceral organs and enlarged storage cells in alveolar and interstitial spaces of the lung. Starting at 10 weeks, animals develop gait ataxia, generalized tremor and gross shaking which lead to a complete paralysis at the age of 20 weeks [11]. A detailed analysis of brain tissue revealed a 10-fold increase of GlcCer in the cerebellum and 2-4-fold increase in other brain regions. Furthermore, glucosylsphingosine a biomarker of GD [12] is 20-117-fold increased in these same regions [13].

So far, 4L/PS-NA mice have been mainly characterized and used for GD research by Grabowski and his team. We therefore analyzed 4L/PS-NA mice for general health and motor deficits over age using standardized behavioral tests. Afterwards, different visceral and neuronal tissues were weighted and visceral organs analyzed for morphological alterations of leukocytes and macrophages. Neuronal tissue was analyzed for GCase activity and substrate levels as well as the progression of neuroinflammation and expression of α-synuclein and calbindin in the cerebellum and hippocampus. Overall, our results show that the phenotype of 4L/PS-NA mice is characterized by a strong visceral and neuronal pathology resulting in severe and progressive motor deficits that can be quantified using different behavioral tests.

## Materials and methods

### Animals

All animals were bred and housed under identical conditions in individually ventilated cages on standardized rodent bedding (Rettenmayer®, Rosenberg, Germany) in the AAALAC-accredited animal facility of QPS Austria. Cotton nestlets (Plexx®, Elst, The Netherlands) were provided as nesting material. The room temperature was kept at approximately 24°C and the relative humidity between 40–70%. Mice were housed under constant light/dark cycle of 12 hours each. Dried pelleted standard rodent chow (Altromin®, Lage, Germany) and normal tap water were available to the animals *ad libitum*. Each individual animal was checked regularly for clinical signs. Animals of both sexes were used. Mice were housed in same sex groups of up to four animals. During weaning, less than 1 mm of the tail tip was cut from each animal and used for genotyping. Actual animal numbers are given in the figure legends. 4L/PS-NA mice with homozygous Gba^V394L/V394L^ mutation (4L) [10], complete prosaposin knockout (PS-) and homozygous prosaposin transgene (NA) resulting in hypomorphic prosaposin levels [11], were bred by mating heterozygous 4L/PS^+/-^NA mice. For all analyses, homozygous 4L/PS-NA mice (called 4L/PS-NA) and control mice with a wild type prosaposin (4L/PS^+/+^NA) of the same age (called control) were used. Heterozygous 4L/PS^+/-^NA breeding pairs were provided by Dr Ying Sun and Dr. Gregory Grabowski, Division of Human Genetics, Children's Hospital Research Foundation and University of Cincinnati College of Medicine, Department of Pediatrics, Cincinnati, OH, USA. For quantification of glucocerebrosidase, glucosylceramide and glucosylsphingosine also 18 week old C57Bl/6RccHsd mice (Envigo, Udine, Italy) were used as additional control.

### Ethics statement

Animal studies conformed to the Austrian guidelines for the care and use of laboratory animals as well as being approved by the Ethics committee of the Styrian government, Austria (ABT13-78Jo199-2017). All efforts were made to minimize suffering.

### Behavioral tests

All behavioral tests were performed during the early phase of the light cycle. Before the start of each behavioral test, animals were habituated to the experimental room for at least 1 hour. Groups were divided according to the phenotypic progression (from onset to late stage), representing relevant time points for compound tests. Animals were allocated to the corresponding experimental group according to their genotype. The following behavioral tests were performed longitudinally in week 8, 12 and 18 in the following order: day 1: Rota Rod and Wire suspension test; day 2: Pasta gnawing test; day 3: Beam Walk test.

The Rota Rod test, Wire suspension test and Pasta gnawing test were conducted as previously described [14, 15]. The Beam walk test was conducted as previously described using a 13 mm square beam for testing [15]. The body weight was measured on testing day 1 using a standard precision scale.

### Tissue sampling

All mice were anesthetized by intraperitoneal injection of 600 mg/kg pentobarbital (Release ®, WDT, Germany). Once animals were deeply anesthetized, mice were transcardially perfused with physiological (0.9%) saline. Thereafter, spleen, liver, lung, thymus and brain were rapidly removed brains hemisected and all tissues weighted using a precision scale. The right hemispheres and all visceral organs were fixed by immersion at 4°C in freshly prepared 4% formaldehyde in phosphate buffer (pH 7.4) for 24 hours. After cryo-conservation in a 15% sucrose/phosphate buffered saline (PBS) solution, tissues were shock frozen in dry ice cooled liquid isopentane. All tissues were then stored at -80°C until used for histological procedures.

Additionally, tissue of naïve 5 and 12 week old animals was sampled for biochemical and histological analyses.

### Glucocerebrosidase measurement

CBE inhibitable GCase activity was measured with a fluorometric assay using 4-Methylumbelliferyl ß-D-glucopyranoside (4-MUG) as substrate. The 4-MUG assay was purchased from Sigma-Aldrich (St. Louis, MO, USA). Brain tissue was homogenized in 10 volumes homogenization buffer (250 mM Sucrose, 10 mM Tris pH 7.5, 0.1% Triton), sonicated on ice and spun at 20.000 xg for 15 min. The supernatants were used for analysis. Samples were mixed 1:1 with 150 mM McIlvaine buffer pH 4.8. Thereafter the substrate mix was added (3.7 mM 4-MUG in 150 mM McIlvaine buffer pH 4.8; 0.1% BSA; 0.4% taurocholate; 0.1% Triton) and incubated for 1 h at 37°C. The reaction was stopped by adding 0.2 M Glycine-NaOH pH 10.8 and fluorescence was detected at Ex WL = 365 nm, Em WL = 445 nm.

Each sample was analyzed in duplicates and a third replicate including 1 mM CBE was used to subtract the GBA1 unspecific signal.

### Glucosylceramide and glucosylsphingosine measurements

Concentrations of glucosylceramide (GlcCer) and glucosylsphingosine (GlcSph) in the extracts from the mouse cerebellum were measured by ultra-high performance liquid chromatography coupled to tandem mass spectrometry (UHPLC-MS/MS) following a slightly modified protocol as described elsewhere (Hamler et al., 2017). Glucosyl(β) ceramide (d18:1/18:0), galactosyl(β) ceramide (d18:1/18:0), glucosyl(β) ceramide-d5, glucosyl(β) sphingosine (d18:1), galactosyl(β) sphingosine (d18:1), glucosyl(β) sphingosine-d5, and galactosyl(β) sphingosine-d5 were purchased from Avanti Polar Lipids (Sigma-Aldrich, St. Louis, MO, USA). Acetonitrile (ACN), methanol (MeOH), formic acid, ammonium formate, all LC-MS grade were purchased from Fisher Scientific (Pittsburgh, PA, USA), dimethyl sulfoxide (DMSO) and all other chemicals were purchased from Sigma-Aldrich. Deionized water (>18 MΩ) was prepared by use of a Direct-Q-3 UV water purification system (Merck Millipore, Darmstadt, Germany).

The dissected brain samples (≈ 50 mg) were mixed with ice-cold water at a ratio 1:10 (w/v). Thereafter, 50 μl of the internal standards (deuterium-labeled GlcSph-d5 and GlcCer-d5, each 4,000 ng/ml) were pipetted, the samples were thoroughly mixed and placed in the ice bath and homogenized by use of a ultrasonic homogenizer Vibra-Cell VCX 130 (Sonics & Materials Inc., Newtown, CT, USA). Immediately after that, 100 μl of the homogenate was pipetted into the separate tubes and 1.3 ml of acetone-methanol (1:1) was added. The tubes were placed in a horizontal shaker and vortexed for 60 min. Thereafter, 1.2 ml of water-MeOH (1:3) was added, the samples were vortexed for 3 min and centrifuged in a refrigerated microcentrifuge Biofuge Fresco (Heraeus Instruments, Hanau, Germany) at 12,000 rpm, 4°C for 15 min. GlcCer and GlcSph were extracted from the brain homogenates by use of solid-phase extraction (SPE) cartridges, BondElut C18 (100 mg) and BondElut Certify (50 mg), respectively, both purchased from Agilent (Santa Clara, CA, USA). The BondElut C18 cartridge was preconditioned with 2 ml of MeOH followed by 2 ml of MeOH-acetone-H2O (67:23:10), the BondElut Certify cartridge was preconditioned with 1 ml of MeOH and 1 ml of water. The supernatant was divided into two equal volumes and slowly loaded onto each SPE column using a low vacuum. The BondElut C18 cartridge was washed with 2 ml MeOH-acetone-H2O (60:25:15) and glucosylceramides and galactosylceramides were eluted with 2 ml of acetone-MeOH (90:10). The BondElut Certify was washed with 2 ml of 0.1 M HCl followed by 2 ml of MeOH and glucosylsphingosine and galactosylsphingosine were eluted with 2 ml of freshly prepared 5% ammonium hydroxide in MeOH. The samples were placed in a vacuum centrifuge miniVac Duo concentrator (Genevac, SP Industries, Warminster, PA, USA) and evaporated to dryness. The samples for GlcCer analysis were reconstituted in 50 μl DMSO and 200 μl ACN-MeOH-H2O (95:2.5:2.5) containing 5 mM ammonium formate and 0.5% formic acid. The samples for GlcSph analysis were reconstituted in 50 μl DMSO and 200 μl ACN-MeOH-H2O (85:7.5:7.5) containing 5 mM ammonium formate and 0.5% formic acid. The UHPLC-MS/MS system included a PAL autosampler (CTC Analytics, Switzerland) operating at 4°C, an Advance UHPLC pump, EVOQ Elite triple quadrupole mass spectrometer (Bruker Daltonics, Billerica, MA, USA) equipped with an electrospray ionization (ESI) source operating in a positive mode, the ESI parameters were as follows: probe gas flow 30, nebulizer gas flow 45, probe temperature 400°C, cone gas flow 15, cone temperature 250°C, the CID gas was Ar set at 1.5 mTorr. The separation of GlcCer from galactosylceramide was achieved on a HALO HILIC column (2.1 x 150 mm, 2 μm particle size) purchased from Advanced Materials Technology (Wilmington, DE, USA), kept at 35°C. The mobile phase included a mixture of acetonitrile (ACN), methanol, water (95:2.5:2.5 v/v) and the final 5 mM ammonium formate and 0.5% formic acid, the flow rate was 0.2 ml/min. The precursor (m/z) and product ions for GlcSph were 462.3 > 282.3 (quantifier ion) and 462.3 > 264.3 (qualifier ion). The separation of GlcSph from galactosylsphingosine was achieved on a HALO HILIC column (2.1 x 100 mm, 2 μm particle size) kept at 35°C. The mobile phase was a mixture of ACN, MeOH and water (85:7.5:7.5), with final 5 mM ammonium formate and 0.5% formic acid, the flow rate was 0.3 ml/min. The precursor (m/z) mass and the product ion of GlcCer was 728.6 and 264.6, respectively, the corresponding m/z mass for the internal standard GlcCer-D5 was 733.6 > 269.6. Typically, 1–3 μl of reconstituted samples for the respective analyte were injected onto the column. Under these conditions the carryover of the method (blank sample injected after a sample with highest concentrations of GlCer and GlcSph was injected) was 0.17% for GlcSph and 0.13% for GlcCer.

### Histology

Frozen brain hemispheres and tissue samples of liver, lung, spleen and thymus were cut in the sagittal or coronal plane at 10 μm thickness. Unless indicated otherwise, a systematic uniform random set of five sections per animal and per tissue sample were used for each immunofluorescent labeling. GFAP + IBA1 and Calbindin D-28k + α-synuclein immunofluorescence incubations of brain sections were conducted as previously described [15]. Sections of visceral organs were treated with a 1 mg/ml sodium borohydride/PBS solution followed by 1% hydrogen peroxide in methanol. To block unspecific labeling, sections were treated with 10% donkey serum for 30 min followed by incubation in primary antibody in 1% donkey serum in PBS overnight at 4°C. Sections were incubated in different highly cross-adsorbed fluorophore-conjugated secondary antibodies all raised in donkey for 1 hour at room temperature. Finally, sections were counterstained with DAPI. Information on the primary antibodies is provided in Table 1. Technical control experiments were routinely performed together with regular experiments.

**Table 1:** List of primary antibodies used for histological evaluations.

Primary antibodies						
Species	Antigen	Clone	Source	Item #	Dilution	stable public ID
Rabbit	GFAP	poly	Dako	Z0334	1:500	AB_10013382
Guinea Pig	IBA-1	poly	Synaptic Systems	234004	1:2,000	AB_2493179
Guinea Pig	Calbindin	poly	Synaptic Systems	214 005	1:1,000	AB_2619902
Mouse	α-synuclein	4D6	abcam	ab1903	1:2,000	AB_302665
Rat	CD45	30-F11	BD Biosciences	550539	1:100	AB_2174426
Rat	F4/80	A3-1	BioRad Laboratories	MCA497R	1:15,000	AB_323279

Mosaic images of the labeled sections were recorded on a fully motorized Zeiss AxioImager Z1 microscope with high aperture lenses, equipped with a Zeiss AxioCam MRm camera and ZEN 2.3 software. Mosaic images were merged and converted to full resolution single channel tif files that were used for quantitative image analysis. The target area was identified by drawing an area of interest (AOI) on the images. Background correction was used if necessary, and the immunofluorescence signal was then quantified by adequate thresholding and morphological filtering (size, shape) to determine the percentage of AOI area covered by immune-positive objects. Once the parameters of the targeted objects had been defined in a test run, the quantitative image analysis ran automatically and macro-based resulting in rater-independent and fully reproducible data. All measurements were performed using ImageProPlus (v6.2) software.

### Statistics

Data analysis was performed in GraphPad Prism 4.03 (GraphPad Software Inc., USA). Graphs show group means and standard error of the mean (SEM). The significance level was set at p < 0.05. Group means were compared using Two-way analysis of variance (ANOVA) with a subsequent *post hoc* test. The utilized statistical tests and exact sample size are mentioned in the figure legends.

### Availability of data and materials

All data generated or analyzed during this study are included in this published article and its supplementary files “S1 Table”.

## Results

### General health and motor deficits of 4L/PS-NA mice over age

**Fig 1:**
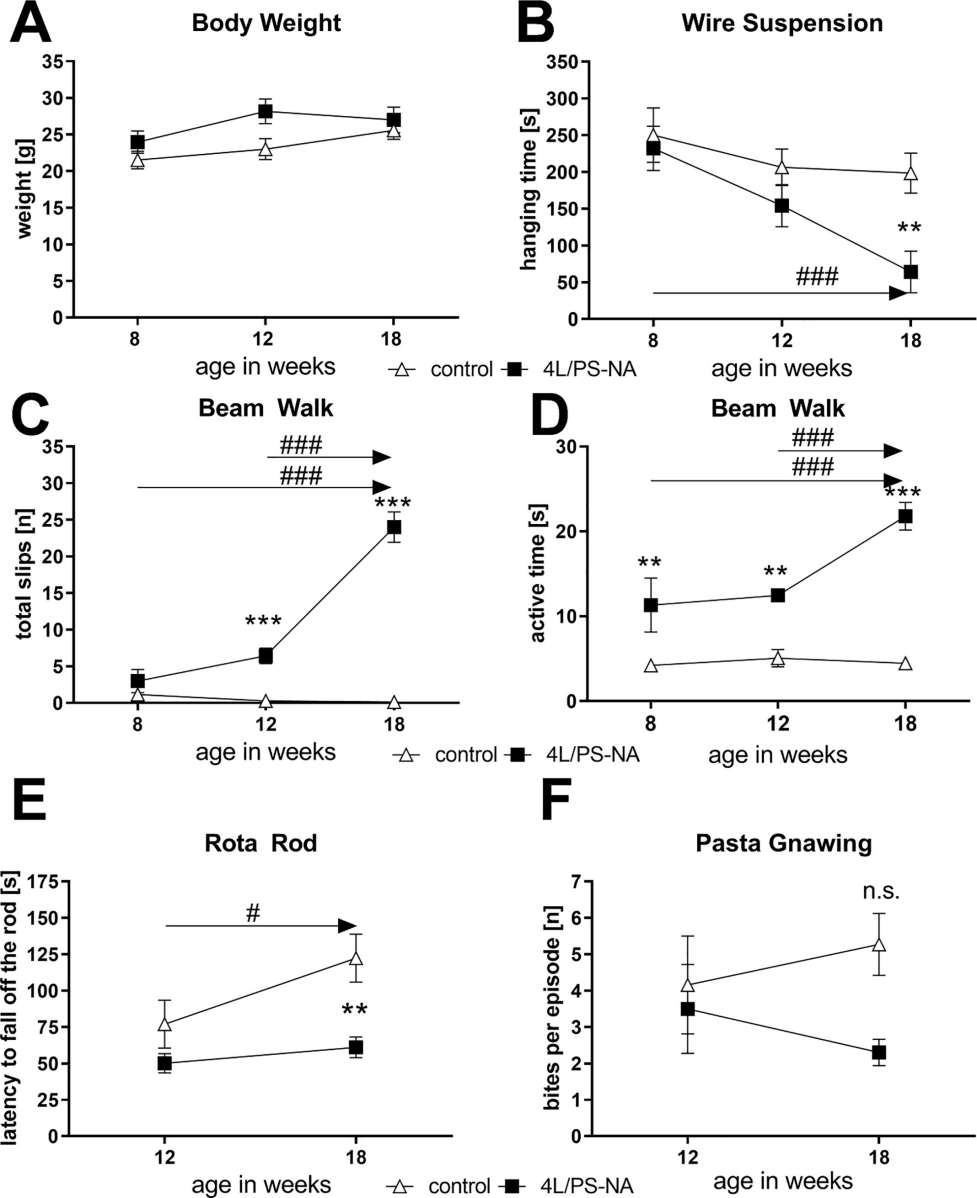
General health and motor deficits of 4L/PS-NA mice over age. Eight, 12 and 18 week old 4L/PS-NA mice and control littermates were analyzed for body weight (**A**), the hanging time in seconds in the wire suspension test (**B**), the total number of slips (**C**) and the active time in the beam walk test (**D**), the latency to fall off the rotating rod of the Rota Rod test (**E**) and the number of bites per episode in the pasta gnawing test (**F**). Two-way ANOVA followed by Bonferroni’s *post hoc* test. n = 7 per group. Mean ± SEM. *differences between genotypes; ^#^differences between age groups; *p<0.05; **p<0.01; ***p<0.001.

To evaluate the health status of 4L/PS-NA mice, animals were weighted over age but no significant changes were observed compared to control animals. Both groups gained weight with increasing age (Fig 1A). The muscle strength of 4L/PS-NA mice was tested in the wire suspension test. Eight week old 4L/PS-NA mice showed a similar muscle strength as control animals but muscle strength significantly decreased over age. Finally, 18 week old 4L/PS-NA mice had a significantly shorter hanging time compared to age matched control animals (Fig 1B). Further analysis of motor deficits in the beam walk test revealed a significant increase of slips in 4L/PS-NA mice compared to controls at 12 week of age. This difference strongly worsened at the age of 18 weeks (Fig 1C). Parallel analysis of the active time in the beam walk test showed that already at the age of 8 weeks 4L/PS-NA mice needed longer to cross the beam compared to control animals. This parameter also worsened over time. 18 week old 4L/PS-NA mice needed more than 4 times the time to cross the beam compared to control animals (Fig 1D). Additional analysis of 4L/PS-NA mice for motor deficits in the Rota Rod test showed a significantly reduced latency to fall off the rod at the age of 18 weeks compared to controls. Furthermore, control animals showed a longer latency to fall off the rod at the age of 18 weeks compared to 12 weeks that was not observed in 4L/PS-NA mice (Fig 1E) suggesting that control animals can improve their performance by learning the motor task over age while 4L/PS-NA mice do not improve. An alternative method to evaluate motor deficits in rodents that does not depend on the animal’s fore- and hind limb muscles is the pasta gnawing test, which is used for analyzing orofacial motor deficits [14]. Analysis of 12 and 18 week old 4L/PS-NA mice in this test revealed no significant changes in the number of bites per episode compared to age matched control animals although a clear trend towards a reduced number of bites per episode in 4L/PS-NA mice was obvious (Fig 1F).

### Progressive tissue weight changes of 4L/PS-NA mice

**Fig 2:**
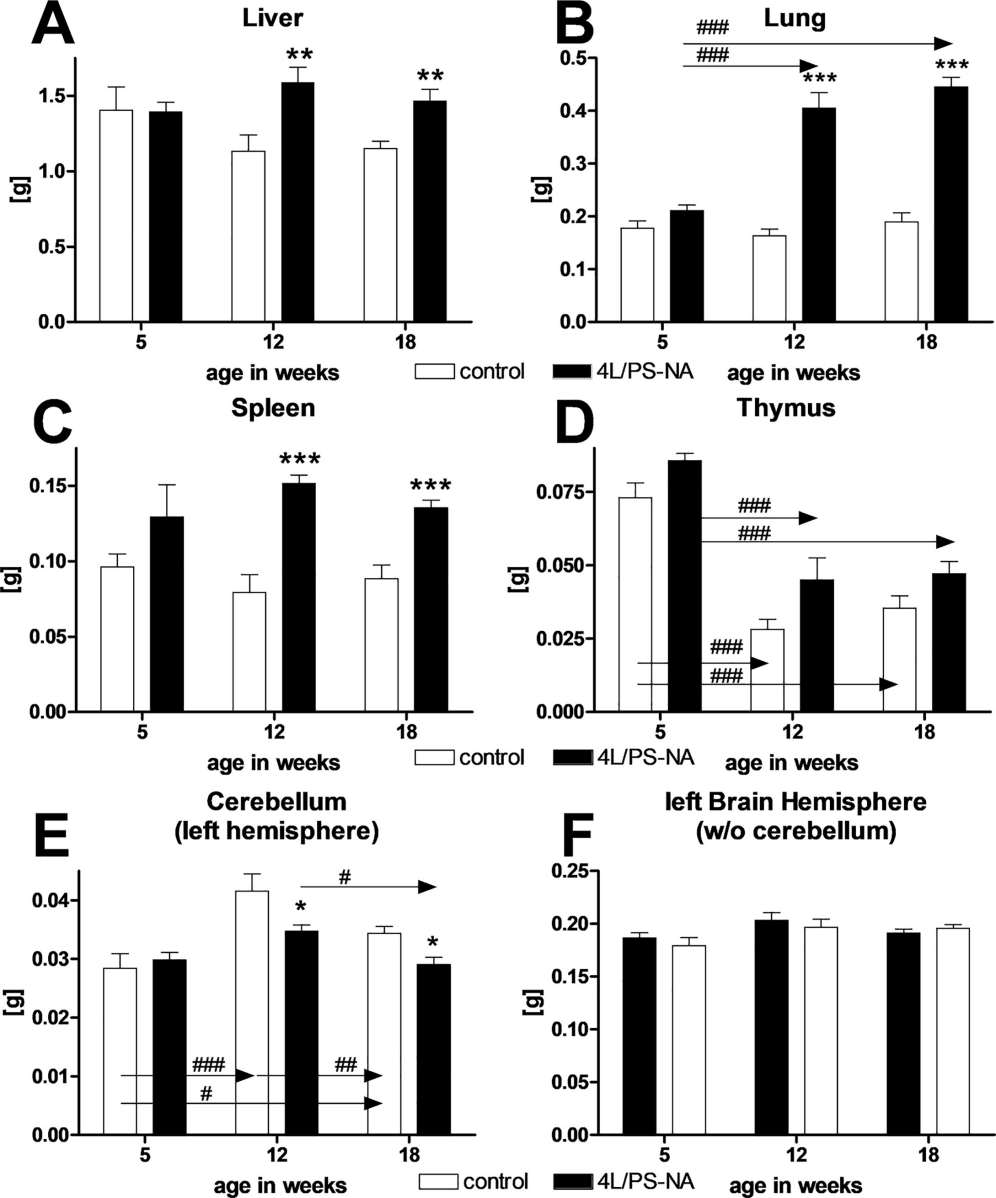
Tissue weight of 4L/PS-NA mice over age. Tissue weight of liver (**A**), lung (**B**), spleen (**C**), thymus (**D**), cerebellum (left hemisphere, **E**) and left brain hemisphere without cerebellum (**F**) were measured at 5, 12, or 18 week of age. Two-way ANOVA followed by Bonferroni’s *post hoc* test. 4L/PS-NA: 5 weeks: n = 6; 12 weeks: n = 7; 18 weeks: n = 15; Control: 5 weeks: n = 5; 12 weeks: n = 7; 18 weeks: n = 16; Mean + SEM. *differences between genotypes; #differences between age groups; *p<0.05; **p<0.01; ***p<0.001.

As already shown in Fig 1A, the total body weight of 4L/PS-NA mice did not change compared to control animals. Since 4L/PS-NA mice are known to display a neuronal as well as non-neuronal phenotype, the weight of different organs of 5, 12 and 18 week old 4L/PS-NA and control mice was measured. The results show that the total weight of liver, lung and spleen of 4L/PS-NA mice was significantly increased at the age of 12 and 18 weeks compared to control animals while no differences were observed at the age of 5 weeks (Fig 2A, 2B and 2C). The total weight of the thymus was highest at 5 weeks of age in 4L/PS-NA as well as control mice and decreased significantly with age but no significant changes were observed between genotypes (Fig 2D).

Weight measurement of neuronal tissue was performed separately for the cerebellum and the remaining brain. While the weight of the cerebellum was decreased in 4L/PS-NA mice compared to control mice from 12 weeks onwards, the weight of the remaining brain did not change over time compared to controls. (Fig 2E and 2F).

It can be summarized that the weight of non-neuronal tissue of 4L/PS-NA mice mostly increased over age while the weight of some neuronal tissue, specifically the cerebellum, decreased over age.

### Non-neuronal phenotype of 4L/PS-NA mice

**Fig 3:**
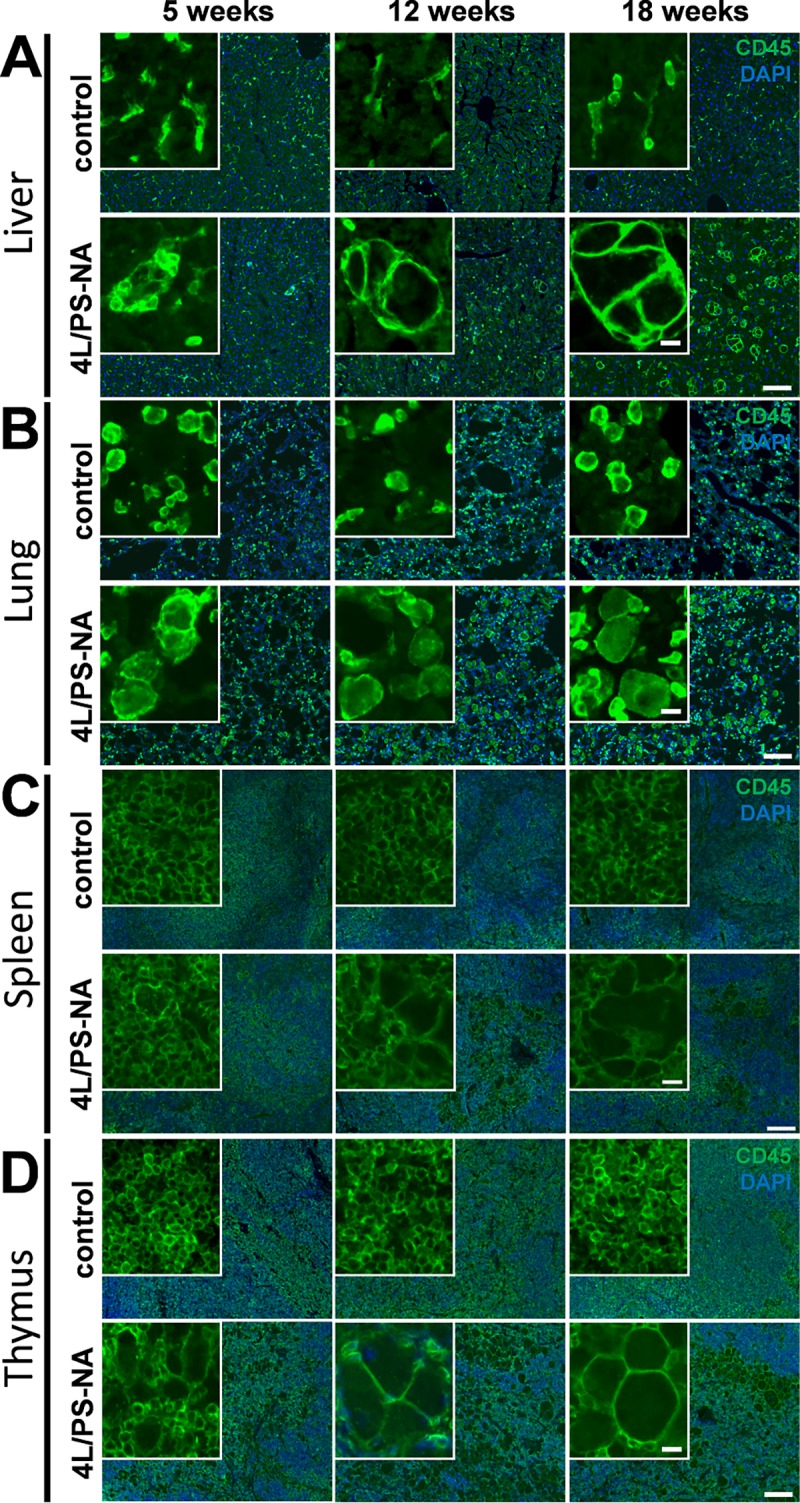
Enlarged leukocytes in visceral organs of 4L/PS-NA mice. The liver (**A**), lung (**B**), spleen (**C**) and thymus (**D**) of 4L/PS-NA mice and control littermates were labeled with CD45 antibody at the age of 5, 12 and 18 weeks; green: CD45; blue: DAPI. Scale bars: Overview: 100 μm, inserts: 10 μm.

To evaluate the visceral pathology of 4L/PS-NA mice, liver, lung, spleen and thymus were immunofluorescently labeled with the specific leukocyte and macrophage markers CD45 and F4/80, respectively. Qualitative analysis of these labelings revealed a progressive increase of leukocyte size in the liver of 4L/PS-NA mice with enlarged cells at the age of 12 weeks. These enlarged cells increased in number and size over age compared to control mice and earlier age groups. The leukocyte size did not seem to further increase at the age of 18 weeks compared to 12 week old animals (Fig 3A). CD45 labeling of leukocytes in the lung of 4L/PS-NA mice showed already many enlarged cells at the age of 5 weeks. Differences between age groups seemed to be minor (Fig 3B). In the spleen of 4L/PS-NA mice, leukocytes were enlarged at the age of 18 weeks compared to control animals. Younger animals barely showed differences in leukocyte size (Fig 3C). Leukocytes in the thymus of 4L/PS-NA mice were strongly enlarged at the age of 5 weeks and progressively inflated until reaching an enormous size at the age of 18 weeks (Fig 3D). The largest leukocytes could thus be observed in liver and thymus.

**Fig 4:**
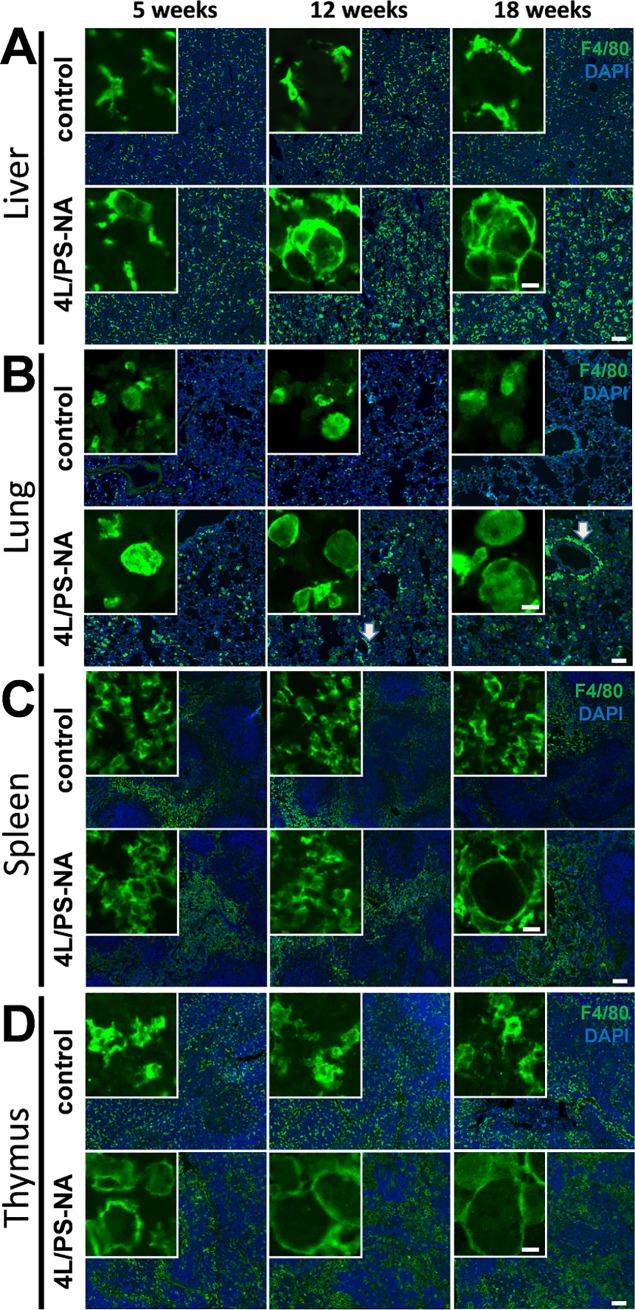
Enlarged macrophages in visceral organs of 4L/PS-NA mice. The liver (**A**), lung (**B**), spleen (**C**) and thymus (**D**) of 4L/PS-NA mice and control littermates were labeled with F4/80 antibody at the age of 5, 12 and 18 weeks; green: F4/80; blue: DAPI. Scale bars: Overview: 100 μm, inserts: 10 μm. Arrows indicate enlarged macrophages surrounding alveoles.

Analysis of macrophage labeling with F4/80 antibody revealed only slightly enlarged macrophages in the liver of 5 week old 4L/PS-NA mice that further progressively inflated (Fig 4A). In the lung of 4L/PS-NA mice, macrophages were slightly enlarged at the age of 5 and 12 weeks and highly enlarged at the age of 18 weeks (Fig 4B). A very prominent feature in the lung was the accumulation of macrophages surrounding alveoli of 4L/PS-NA mice as indicated by white arrows in Fig 4B. Macrophages in the spleen were highly enlarged in 18 week old 4L/PS-NA compared to control mice. The spleen of younger 4L/PS-NA mice did not seem to contain enlarged macrophages (Fig 4C). Labeling of macrophages in the thymus of 4L/PS-NA mice revealed highly inflated macrophages already at the age of 5 weeks that progressively increased in size (Fig 4D). The largest macrophages could thus be observed in the spleen and thymus.

### Neuronal phenotype of 4L/PS-NA mice

To evaluate the enzyme activity of glucocerebrosidase and measure the substrates glucosylceramide and glucosylsphingosine, neuronal tissue of 5, 12 and 18 week old 4L/PS-NA mice, control animals as well as C57Bl/6 mice was analyzed by 4-MUG assay, UHPLC-MS/MS and UPLC-TOF, respectively.

**Fig 5:**
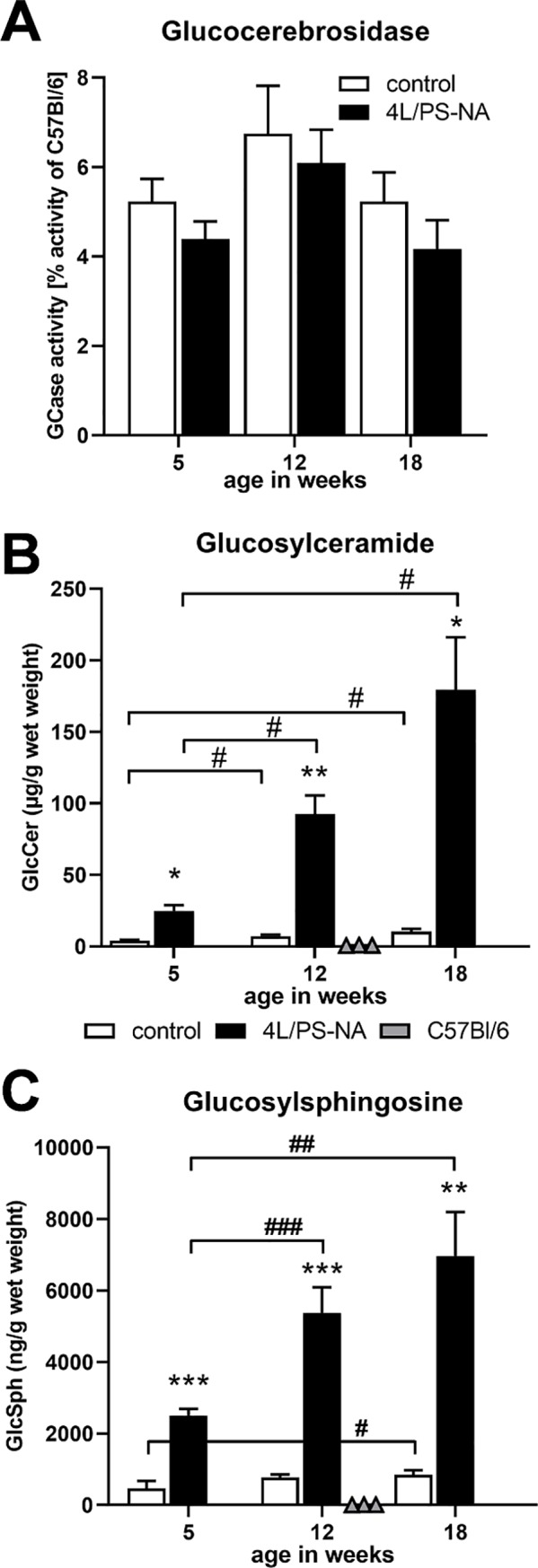
Quantification of glucocerebrosidase, glucosylceramide and glucosylsphingosine in 4L/PS-NA mice over age. The cortex of 5, 12 and 18 week old 4L/PS-NA mice, control animals as well as C57Bl/6 mice was analyzed for glucocerebrosidase activity by 4-MUG assay **(A)**. Whole brain homogenates of 5, 12 and 18 week old 4L/PS-NA mice, control animals as well as C57Bl/6 mice were analyzed for glucosylceramide **(B)** and glucosylsphingosine **(C)** levels in μg/g wet weight **(B)** or ng/g wet weight **(C)**. Two-way ANOVA followed by Bonferroni’s *post hoc* test. Values of C57Bl/6 mice (triangles in **B** and **C**) were excluded from all statistical analyses; n = 5 for 4L/PS-NA and control animals; n = 3 for C57Bl/6 mice. Mean + SEM. *differences between genotypes; ^#^differences between age groups; *p<0.05; **p<0.01; ***p<0.001.

Analysis of the GCase activity revealed that 4L/PS-NA mice and also control animals presented a drastically reduced signal of only approximately 6% compared to C57Bl/6 mice (Fig 5A). This result was independent of the animal’s age.

Substrate measurements in brain homogenates showed significantly increased glucosylceramide and glucosylsphingosine levels as early as 5 week of age (Fig 5B and 5C). At this early age glucosylceramide levels were already about 6-fold higher, while glucosylsphingosine levels where about 5-fold higher compared to controls. Levels of both substrates significantly increased over age in 4L/PS-NA and control animals. While glucosylceramide levels in control animals increased from week 5 to 18 about 2.5-fold, levels of 4L/PS-NA mice were about 7.5-fold increased. This effect was weaker for glucosylsphingosine levels. In control animals the substrate increased from week 5 to 18 about 1.8-fold and in 4L/PS-NA mice about 2.7-fold. For both substrate measurements tissue of 12 week old C57Bl/6 mice was evaluated as additional control since the control littermates of 4L/PS-NA mice are still genetically altered (Fig 5B and 5C). The analysis of C57Bl/6 mouse tissue showed only a minor signal for both substrates (Fig 5B and 5C, grey triangles).

**Fig 6:**
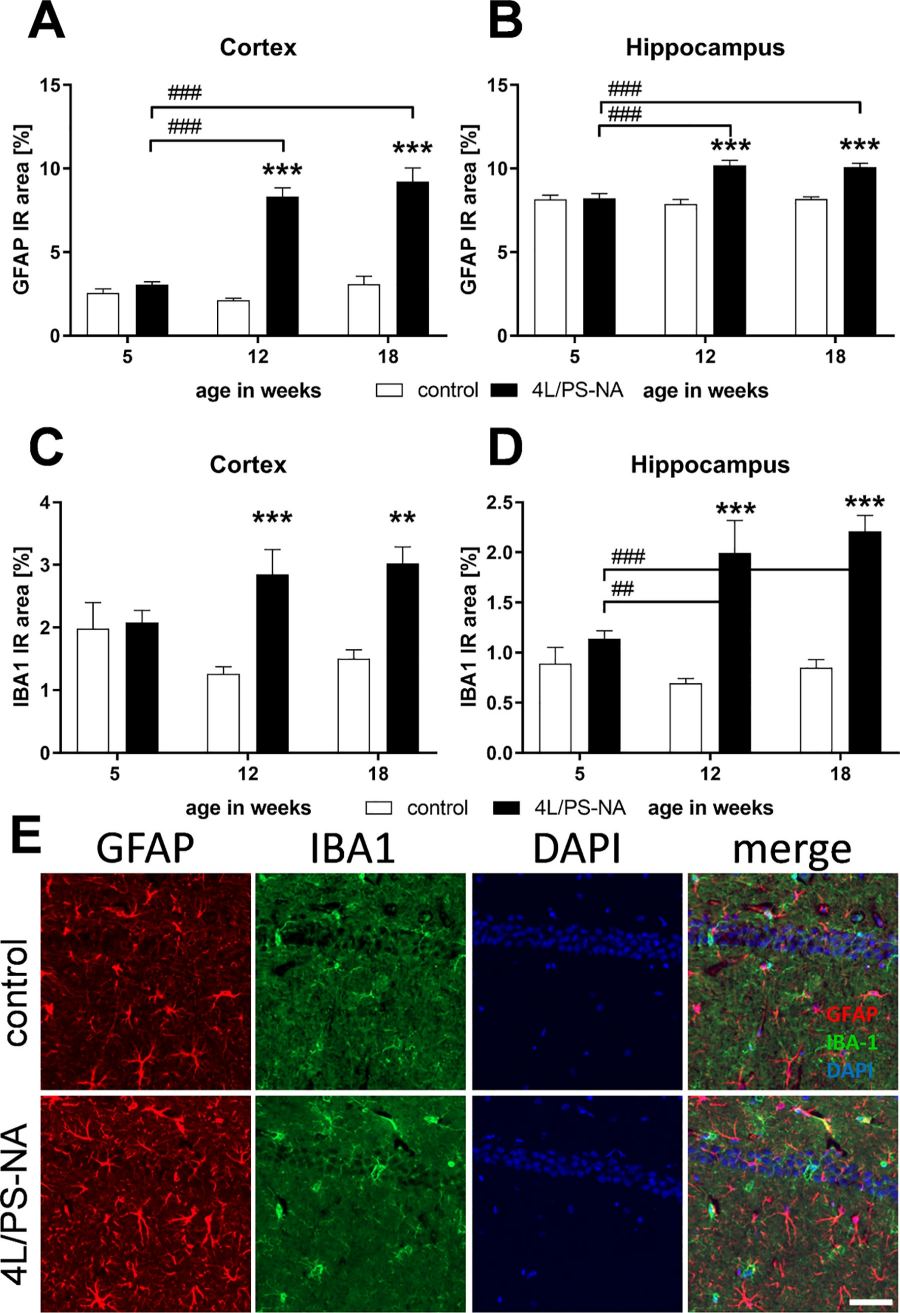
Quantification of astrocytosis and activated microglia in 4L/PS-NA mice over age. The cortex (**A,C**) and hippocampus (**B,D**) of 4L/PS-NA mice was analyzed for GFAP (**A,B**) and IBA1 (**C,D**) immunoreactive area (IR) in percent at the age of 5, 12 and 18 weeks compared to control littermates. Two-way ANOVA followed by Bonferroni’s *post hoc* test. n = 5 per group. Mean + SEM. *differences between genotypes; ^#^differences between age groups; **p<0.01; ***p<0.001. **E**: Representative images of GFAP, IBA1 and DAPI labeling of the hippocampal CA1 region in 18 week old 4L/PS-NA and control mice. Scale bar: 50 μm.

A common neuronal pathology in GD is neuroinflammation [16, 17]. The cortex and hippocampus of 5, 12 and 18 week old 4L/PS-NA mice were thus analyzed for astrocytosis and activated microglia using GFAP- and IBA1-specific antibodies, respectively. Quantification of the immunoreactive area (IR area) showed a similar expression of GFAP and IBA1 labeling in both brain regions. The IR area of both neuroinflammation markers was significantly increased in 12 and 18 week old 4L/PS-NA mice compared to controls and to 5 week old 4L/PS-NA mice. In 5 week old animals, no significant changes were observed (Fig 6A–6D). Representative images of GFAP and IBA1 labeling of the hippocampal CA1 region in 18 week old 4L/PS-NA and control mice are shown in Fig 6E.

**Fig 7:**
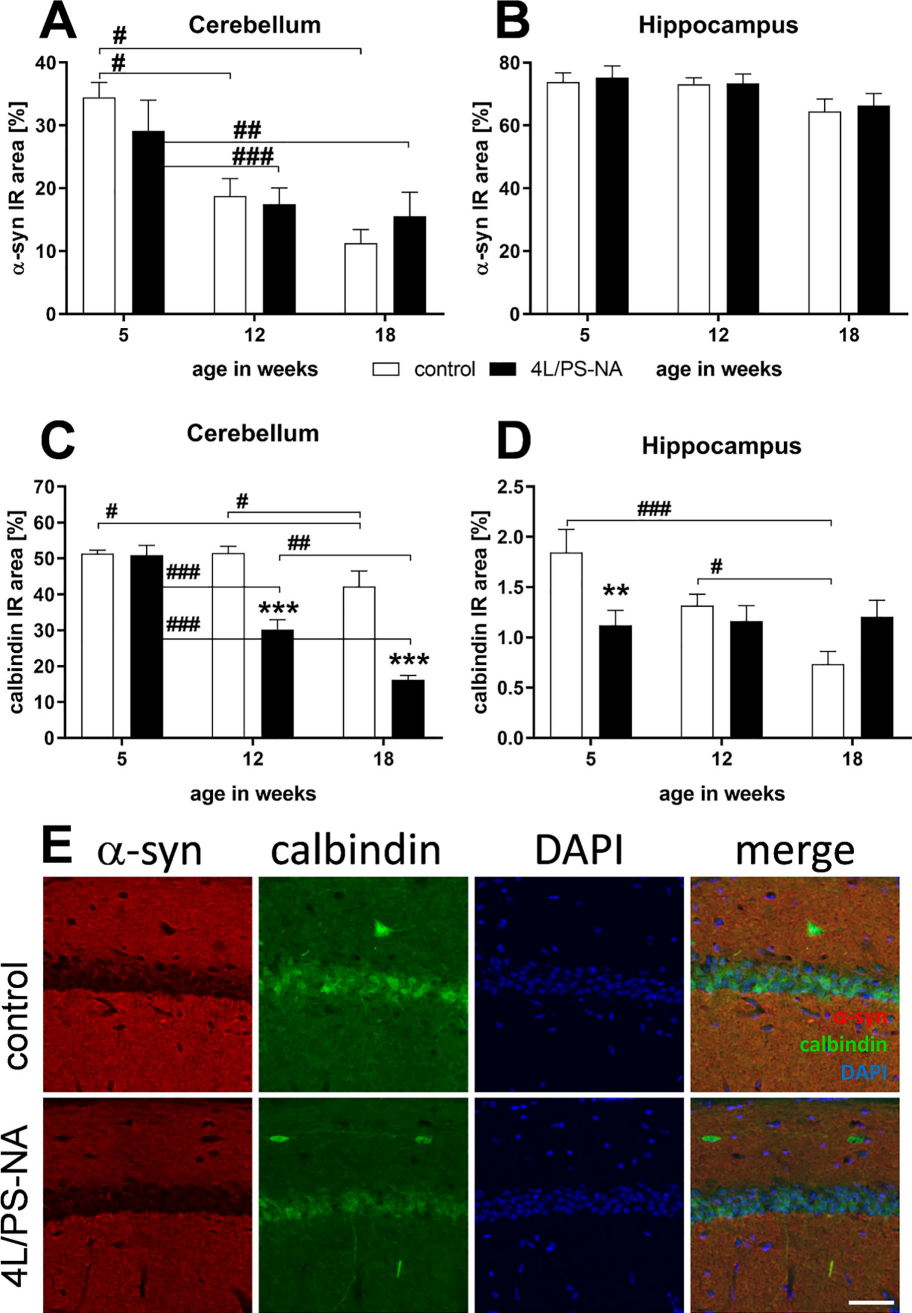
Quantification of α-synuclein and calbindin in 4L/PS-NA mice over age. The cerebellum (**A,C**) and hippocampus (**B,D**) of 4L/PS-NA mice was analyzed for pan α-synuclein (**A,B**) and calbindin (**C,D**) immunoreactive area (IR) in percent at the age of 5, 12 and 18 weeks compared to control littermates. Two-way ANOVA followed by Bonferroni’s *post hoc* test. n = 5 per group. Mean + SEM. *differences between genotypes; ^#^differences between age groups; *p<0.05; **p<0.01; ***p<0.001. **E**: Representative images of pan α-synuclein, calbindin and DAPI labeling of the hippocampal CA1 region in 5 week old 4L/PS-NA and control mice. Scale bar: 50 μm.

GD is characterized by an increased aggregation of α-synuclein protein, linking the disease to synucleinopathies [18–20]. α-synuclein was therefore labeled in the cerebellum and hippocampus of 5, 12 and 18 week old 4L/PS-NA mice and the IR area compared to age matched controls. The α-synuclein IR area was highest in the cerebellum and hippocampus of 5 week old control and 4L/PS-NA animals. In the cerebellum α-synuclein levels decreased over age in both groups without significant differences between genotypes (Fig 7A). In the hippocampus, only a minor decrease of α-synuclein levels over age could be observed with the here applied method (Fig 7B).

Labeling the cerebellum with an antibody against the calcium binding protein calbindin showed highly decreased calbindin levels in 4L/PS-NA mice at the age of 12 and 18 weeks compared to control animals. The decrease in the calbindin signal worsened over age in 4L/PS-NA mice while in control animals the signal decreased only in 18 month old animals (Fig 7C). In the hippocampus on the contrary, calbindin labeling stayed constant over age in 4L/PS-NA mice while the levels were high in control animals at the age of 5 weeks and progressively decreasing over age. This effect resulted in significantly decreased calbindin levels in 5 week old 4L/PS-NA mice compared to control animals of the same age (Fig 7D). Representative images of α-synuclein and calbindin labeling in the hippocampal CA1 region of 5 week old 4L/PS-NA and control mice are shown in Fig 7E.

### Cerebellar alterations of 4L/PS-NA mice

**Fig 8:**
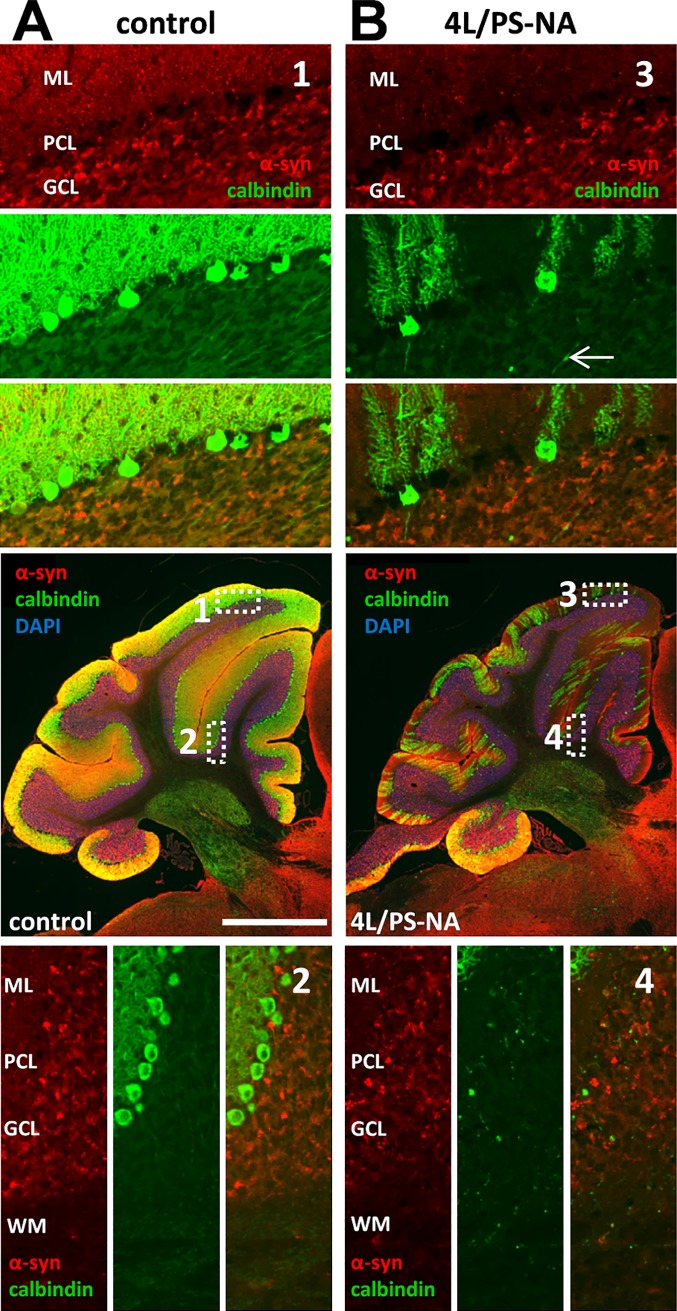
Pathological alterations in the cerebellum of 4L/PS-NA mice. The cerebellum of 18 week old 4L/PS-NA mice (**B**) and control littermates (**A**) were labeled with antibodies against α-synuclein (red) and calbindin (green) and nuclei were visualized with DAPI (blue). Scale bar: 1,000 μm. 1, 2, 3 and 4 show magnifications of areas indicated in the overview images. DAPI staining is not shown in higher magnification images. arrow: Purkinje cell axon; ML: molecular layer; PCL: Purkinje cell layer; GCL: granule cell layer; WM: white matter.

Since the tissue weight and the calbindin signal in the cerebellum of 4L/PS-NA mice decreased over age (Figs 2E and 7C), the pathological features of the cerebellum were analyzed in more detail. Sagittal sections of the cerebellum of 4L/PS-NA and control animals were immunofluorescently labeled with antibodies against α-synuclein and calbindin and nuclei were stained with DAPI. Qualitative analyses revealed a strongly disturbed Purkinje cell layer with a decreased number of Purkinje cells in 4L/PS-NA mice. The molecular layer containing i.a. the dendritic tree of Purkinje cells was thus strongly striped and scattered in 4L/PS-NA mice (Fig 8B). In the granule cell layer single Purkinje cell axons labeled by calbindin could be observed (arrow head in Fig 8B). α-synuclein labeling was almost limited to the granule cell layer in 4L/PS-NA mice.

All experiments were not sex-specifically analyzed since group sizes would have been too small for statistical analyses. No obvious differences between male and female 4L/PS-NA mice could be observed.

## Discussion

### Motor deficits of 4L/PS-NA mice

Here we show for the first time the results of a standardized longitudinal behavioral test battery of 4L/PS-NA mice that are summarized in Table 2. Previously the phenotype of 4L/PS-NA was described to start after the age of 10 weeks presenting gait ataxia, gross shaking and as first symptom a lack of rear limb control that at the age of approximately 20 weeks ends in a complete paralysis [11]. These results seem to be based on cage side observation without quantification or statistical analyses. Here we could show that first signs of motor deficits are already quantitatively measurable starting at 8 week of age and that the observed phenotype progresses over age. With this behavioral test battery it was thus possible to analyze 4L/PS-NA mice for symptoms that are similarly described in GD patients like abnormal gait and bulbar dysfunctions like respiratory problems, dysphagia and altered speech [21–25].

**Table 2:** Summary of health and behavioral changes in 4L/PS-NA mice compared to control animals.

		age in weeks	effect
**Health and Behavior**	Body Weight	18	-
	Wire suspension	18	↓
	Beam Walk	8	↓
	Rota Rod	18	↓
	Pasta Gnawing	18	-

Age of symptom onset is indicated in weeks. ↓: decrease of performance;—up to the indicated age no significant changes observed.

### Visceral alterations in 4L/PS-NA mice

Analysis of 4L/PS-NA mouse organs revealed a weight increase of most visceral organs. Specifically the liver and spleen of 4L/PS-NA mice showed a strong increase in weight, a pathology that is commonly observed in GD patients [26]. In 4L/PS-NA mice an organ weight increase of about 1.5-fold could be observed, a result that is relatively minor compared to GD patients that can present spleen sizes that are in most patients with a still intact spleen more than 5 times the size compared to healthy controls [26]. The weight of the lung of 4L/PS-NA mice doubled compared control animals, suggesting a strong effect of the genetic alterations on pulmonary pathology. Pulmonary problems are mostly described as rare in GD patients, although a *post mortem* study revealed pulmonary involvement in all analyzed cases [27]. The weight of the thymus of 4L/PS-NA mice did not significantly change, a finding that seems to fit to the literature, since the thymus is barely mentioned in GD case studies. The observed progressive decrease in thymus weight independent of the genetic alteration is a well-known physiological loss of organ mass, called involution, and associated with a decrease in thymus activity (see review [28] for overview). Evaluation of macrophages and leukocytes in these four analyzed visceral organs revealed a very early onset of leukocyte and macrophage enlargement in lung and thymus, while this effect was delayed in liver and latest seen in spleen. These results are in line with analyses by Sun and colleagues [11], but morphological changes do not relate to the observed tissue weights. Additionally, the strongest phenotype observed in GD patients relates to liver and spleen, both organs that in the mouse model show a delayed symptom onset compared to the lung and thymus that are in patients not that obvious or not at all affected [26]. For an overview of the visceral phenotype see Table 3.

**Table 3:** Summary of visceral changes in 4L/PS-NA mice compared to control animals.

	Liver		Spleen		Lung		Thymus	
	age in weeks	effect	age in weeks	effect	age in weeks	effect	age in weeks	effect
Weight	12	↑	12	↑	12	↑	18	-
Leukocytes	12	↑	18	↑	5	↑	5	↑
Macrophages	5	↑	12	↑	5	↑	5	↑

Age of symptom onset is indicated in weeks. ↑: increase of pathology;—up to the indicated age no changes observed.

### Neuronal alterations in 4L/PS-NA mice

Analysis of tissue weight in the brain revealed a weight loss for the cerebellum but not for the whole brain hemisphere, suggesting that the loss is area-specific. A corresponding disorganization and reduction of Purkinje cells could be observed by immunofluorescent labeling as already previously shown by Sun and colleagues [11]. Measurement of GCase activity revealed no significant changes between 4L/PS-NA and control animals. Since both strains contain the homozygous Gba^V394L/V394L^ mutation it was expected that the GCase activity is similarly reduced in both groups. Our results thus show that the Gba^V394L/V394L^ mutation is not sufficient to cause a GD-related phenotype in mice since control mice do not show any neuronal or visceral alterations, a result that is in agreement with previous work by Xu and colleagues [10]. Only in combination with the prosaposin or saposins C reduction, animals present a disease-relevant neuronal and visceral phenotype [11, 29].

Quantification of GlcCer and GlcSph in the cerebellar brain extracts from 4L/PS-NA mice revealed dramatic and age-dependent increases both in the GlcCer and in GlcSph levels. Thus, GlcCer concentrations increased about 18 times in the cerebellum of 18 week old 4L/PS-NA mice as compared to controls at the same age, which is comparable to the reported 23 times increase in 20 week old 4L/PS-NA mice [30]. Even a higher, 32.6-fold increase in the levels of GlcCer d18:1/18:0 in the cerebellum of 18 week old 4L/PS-NA mice was reported [31]. A possible reason for the difference in the estimated GlcCer ratio between the 4L/PS-NA and control mice could be a lower estimated level of GlcCer in 4L/PS-NA mice, since the mean cerebellar GlcCer levels in the 4L/PS-NA mice reported by [31] were about 145 μg/g, which is similar to the levels of 180 μg/g wet weight measured in our study. The highest levels of GlcSph in the cerebellum of 18 week old 4L/PS-NA mice was in average 8.4 times higher as compared to control mice, which is well in agreement to the reported 9 times higher GlcSph in the 4L/PS-NA of 20 week old mice [30]. Interestingly, almost the same (8.3) fold increase in GlcSph was measured already in 5 week old 4L/PS-NA mice, as compared to only 6.4 times increase in GlcCer levels. This indicates that the abnormal levels of GlcSph in the brain of 4L/PS-NA mice occur already at juvenile age and the ratio to the GlcSph levels in the controls remains constant over the following 3 to 4 months. This finding suggests that GlcSph may serve as a more reliable biomarker to study therapeutic interventions in the 4L/PS-NA mouse model of GD compared to GlcCer, Indeed, plasma GlcSph was recently proposed as a more sensitive and specific biomarker for primary diagnostic and follow-up monitoring in GD patients [12, 32, 33].

Already in 2004, Wong and colleagues showed astrocytosis in hippocampal *post mortem* tissue of GD patients [16]. Furthermore, Burrow and colleagues could show activated microglia/macrophages by CD68 labeling in the cerebellum, basal ganglia and hippocampus of a GD patient [34]. Evaluations of neuroinflammation in different GD mouse models showed repeatedly strong inflammatory processes [35–37]. In these mouse models, increased proinflammatory markers as well as activated microglia and astrocytosis could be verified and linked to neuronal loss in distinct brain regions [35, 36]. Here we show strong neuroinflammation using activated microglia and astrocytosis marker in the cortex and hippocampus of 12 week old 4L/PS-NA mice. In combination with results of other GD mouse models these results suggest that neuroinflammation is a common and solid phenotype of these animal models. The phenotype should thus be well-suited to evaluate the neuronal effect of new compounds that are expected to influence inflammation and other neuronal phenotypes of the disease.

In recent years *GBA1* mutations were repeatedly linked to Parkinson’s disease, suggesting that Parkinson’s disease patients present *GBA1* mutations significantly more frequently than healthy control groups [38–40]. Additionally, it was shown that *GBA1* mutations can influence the aggregation of α-synuclein in *in vitro* experiments [41] by its substrate GlcSph [42]. We therefore analyzed 4L/PS-NA mice for α-synuclein in the cerebellum and hippocampus resulting in no major changes between age groups. This result contradicts with previous results of α-synuclein aggregations in 4L/PS-NA mice [13] and the effect of GBA1 downregulation on α-synuclein accumulation [20, 43]. Observed differences might depend on analyzed brain regions or even more likely the used primary α-synuclein antibody. Future studies are thus needed to evaluate if 4L/PS-NA mice indeed show increased α-synuclein accumulations. Such phenotype would allow the use of the mouse model for analysis of GBA-associated Parkinson’s disease.

Histological analyses of the 4L/PS-NA mouse cerebellum and hippocampus revealed strong quantitative changes in calbindin levels compared to control animals. Cerebellar changes and corresponding neuronal symptoms are common in GD patients, especially children [44, 45]. In an animal model with a mutation of the saposin C domain of the prosaposin gene a reduction of Purkinje cells was observed, suggesting that this phenotype depends on the hypomorphic prosaposin levels of 4L/PS-NA mice [46, 47]. For a summary of neuronal changes in 4L/PS-NA mice see Table 4.

**Table 4:** Summary of quantitative neuronal changes in 4L/PS-NA mice compared to control animals.

	Cerebellum	Whole brain	Cortex	Hippocampus
	age in weeks	effect	age in weeks	effect	age in weeks	effect	age in weeks	effect
Weight	12	↓	18	-	N/A	N/A	N/A	N/A
Gcase	N/A	N/A	N/A	N/A	18	-	N/A	N/A
GlcCer	N/A	N/A	5	↑	N/A	N/A	N/A	N/A
GlcSph	N/A	N/A	5	↑	N/A	N/A	N/A	N/A
Astrocytosis	N/A	N/A	N/A	N/A	12	↑	12	↑
act. Microglia	N/A	N/A	N/A	N/A	12	↑	12	↑
α-Synuclein	18	-	N/A	N/A	N/A	N/A	18	-
Calbindin	18	-	N/A	N/A	N/A	N/A	18	↑

Age of symptom onset is indicated in weeks. ↑: increase of pathology; ↓: decrease of pathology;—up to the indicated age no significant changes observed; N/A: not analyzed.

### Comparison of 4L/PS-NA mice with other mouse models of Gaucher disease

Researchers struggled for a long time to develop proper mouse models of GD. First attempts were made by developing a mouse model with a targeted disruption of the GBA gene. This intervention caused a strong reduction of the GCase activity but also a very early death of these animals at postnatal day 1 [7, 8]. Several research groups than tried to introduce point mutations into the GBA gene to induce GD. Introduction of the RecNcil mutation caused a strong reduction in GCase activity and accumulations of different substrates in the liver and brain. The same researchers also tested the effect of the GBA^L444P^ mutation in mice that caused a weaker GCase reduction and no substrate accumulations. Interestingly, both models died within 48 hours after birth [9]. Further analysis of different GBA mutations caused either a neonatal lethality (GBA^N370S^) [10], short survival of 4 weeks with only a minor inflammatory phenotype [48] or no gross phenotype at all (GBA^D409H^, ^D409V^, ^V394L^) [10]. Another attempt was performed by completely deleting the prosaposin gene that was either neonatally fatal or caused a very rapidly progressing neurological and hepatic phenotype with a maximal survival time of 38 days [6]. A partial rescue of the prosaposin deletion as performed by Sun and colleagues resulted in an extended survival time, depending on the rate of rescue of up to 230 days but in parallel causing an almost complete loss of phenotype [5]. From these studies it was concluded that prosaprosins/saposins need to be reduced below a specific threshold to induce a GD-relevant phenotype in mice [5]. In the here characterized GD mouse model a new approach of reduced prosaposin/saposins expression combined with homozygous *Gba1*^V394L^ was therefore used, resulting in a strong visceral and neuronal phenotype and a survival time that provides modifiable disease symptoms and an adequate time window for treatment studies, respectively.

### Comparison of 4L/PS-NA and human GD phenotype

When comparing the symptoms observed in GD mouse models with the human disease, it becomes obvious that most published models as described above mimic the type 2 GD by having a very short survival time as described for this acute neuronopathic disease form. 4L/PS-NA mice show many of the visceral and neuronal symptoms observed in type 2 and 3 GD but with an extended survival time suggesting that these mice represent type 3 GD, the chronic neuronopathic disease form. Due to the strong similarities in geno- and phenotype of 4L/PS-NA mice compared to type 3 GD, a high translational value for 4L/PS-NA mice can be concluded.

## Conclusions

The here presented detailed characterization of 4L/PS-NA mice shows for the first time a quantification of motor deficits over age. Visceral organs like liver, lung and spleen of 4L/PS-NA mice present a strong increase in weight combined with highly enlarged leukocytes and macrophages. The neuronal phenotype of 4L/PS-NA mice is characterized by a reduced glucocerebrosidase activity and at the same time highly increased glucosylceramide and glucosylsphingosine substrate levels. Additionally, a strong neuroinflammation as indicated by astrocytosis and activated microglia in the cortex and hippocampus of 4L/PS-NA mice was found. A detailed analysis of cerebellar tissue revealed tissue weight loss and decreased calbindin levels that are combined with a strong Purkinje cell loss in 4L/PS-NA mice while α-synuclein levels did not change compared to control animals. These results show that 4L/PS-NA mice present a phenotype that is highly comparable to the pathological alterations described for GD patients. 4L/PS-NA mice have therefore a high translational value for the development and analysis of new compounds against this devastating lysosomal storage disease.

## Supporting information

S1 Table Raw data of Figs 1, 2, 5, 6 and 7. Data sheets show raw data of Fig 1A–1F, Fig 2A–2F, Fig 5A–5C; Fig 6A–6D and Fig 7A–7D. All other data are included in the main manuscript.
(XLSX)

## Abbreviations

AAALAC: Association for Assessment and Accreditation of Laboratory Animal Care
ANOVA: Analysis of variance
CD45: cluster of differentiation 45
DAPI: 4’,6-Diamidin-2-phenylindol
ERT: enzyme replacement therapy
GCase: glucocerebrosidase
GD: Gaucher disease
GlcCer: glucosylceramide
GFAP: Glial fibrillary acidic protein
IBA1: ionized calcium-binding adapter molecule 1
IR: immunoreactive
SRT: substrate reduction therapy
